# Fabrication of plane-type axon guidance substrates by applying diamond-like carbon thin film deposition

**DOI:** 10.1038/s41598-023-35528-3

**Published:** 2023-05-25

**Authors:** Masahito Ban, Jing Chen

**Affiliations:** 1 Department of Applied Chemistry, Faculty of Fundamental Engineering, Nippon Institute of Technology, 4-1, Gakuendai, Miyashiro, Minami-Saitama, Saitama, 345-8501 Japan; 2 Environmental Symbiotic System Major, Nippon Institute of Technology, 4-1, Gakuendai, Miyashiro, Minami-Saitama, Saitama, 345-8501 Japan; 3 Present Address: WORLD INTEC CO., LTD., Kobe, Japan

**Keywords:** Lab-on-a-chip, Axon and dendritic guidance, Biomedical materials, Biomedical engineering

## Abstract

This research aims to fabricate plane-type substrates for evaluating the axon behaviors of neuronal cells in vitro toward the development of brain-on-chip models by applying the functions of diamond-like carbon (DLC) thin film deposition, which helped to eliminate the costly and time-consuming lithography process by utilizing a shadow mask. The DLC thin films were partially deposited on stretched polydimethylsiloxane (PDMS) substrates covered with a metal mask by the plasma chemical vaper deposition method, and using the substrates culture teats with human neuroblastoma cells (SH-SY5Y) were performed. Three patterns of interconnection structures of axons were created on the substrates with disordered and regular linear wrinkle structures with several μm size formed by the depositions. The patterns were characterized by the structure that the aggregations of axons formed on the linear DLC thin film deposited areas were separately placed in regular intervals and connected each other by plenty of axons, which were individually taut in a straight line at about 100 to over 200 μm in length. The substrates expected of uses for evaluation of axon behaviors are available without fabricating guiding grooves by conventional soft lithographic methods requiring multiple stages and their treating times.

## Introduction

Evaluation in vitro of cells of the nervous system is one important and occasionally essential step for not only fundamental cellular processes but also biological applications, cytotoxicity assessments and medical attentions in various diseases relating to the central and peripheral nervous system, and spinal cord. The network of nerves constructed in vitro can also be expected to be a useful assist to simplify the complicated central nervous system, and to evaluate some functions separately. Brain-on-chip models, which have been derived from microfluidic devices, are the platform systematically developing the ideas, and can realize the experiments and evaluations, for which the uses of living organisms are difficult or not permitted^1–3^. The technology is expected to serve as alternatives to a variety of neurological functions of the human brain, and has been under intense study for the uses in medical fields, e.g., from understanding the physiology and disease mechanisms to pharmacokinetics, pharmacodynamics and toxicology^4–6^. One of more important tasks in the realization of brain-on-chip models is probing axons independently from cell bodies, and therefore the techniques for positioning somata and guiding axons are required.

The configuration and used materials, and the fabrication process of the brain-on-chips have been based on mainly the technology in microfluidic chips^1^. Therefore, they are composed chiefly of an underlying substrate formed hundreds of micrometers size of microstructures such as channels, chambers, etc. on the surface, and a top cover, being both made of mainly polydimethylsiloxane (PDMS) and glass. They have been fabricated by almost same process as microfluidic chips, based on softlithography techniques^7^, and a variety of 2- and 3-dimensional (2D and 3D) designs of the chips have been created. To fabricate 2D design chips, a microcontact printing (μCP) method has been often used to change the surface property of designed specified areas, which are achieved by being contacted the convex side of the stamp fabricated with photolithography. Several research applying the μCP method to neuronal cell guidance relating to brain-on-chip models can be found^8–10^. Offenhausser et al.^9^ presented the guiding of neurite outgrowth along the micropatterned lines prepared by the μCP method, for which a blend of extracellular matrix molecules or concanavalin A was stamped as the adhesive grid pattern on cell-repellent background surface treated by poly(styrene) or poly(ethyleneglycol). Moreover, various micrometer-size components essential to 3D design chips, such as microchannels, microgrooves, microchambers, microwells and so on, have been generally formed by means of softlithographic methods, in which a master mold made using mainly photolithography was transferred to a polymeric material, PDMS in many cases. In 3D design chips, three-dimensional constraints such as microchannels, microchambers and microgrooves have been often utilized as tools for guidance of axons. These components were built up mainly at the sizes of a few to tens of micrometers in width, and one to several axons grew through the tracks^11–15^. Also, 3D design chips for isolating axons independently from cell bodies were invented, demonstrating that the compartments for axons and somata were constructed as microchannels, and separated by a partition with a lot of embedded microgrooves, in which axons were passed through^11,12^. These brain-on-chip models equipped with the individual grown axons have been proposed for investigating central nervous system axonal injury^12,15^. Moreover, research on nano-sized grooves, being around hundreds of nanometers in width, were performed, reporting that the nano pattern surfaces had the effects on the anisotropy of axonal outgrowth, neuronal cell activity and network connectivity^16,17^. While, Millet et al.^18^ reported that the control in guiding neuron development and network formation was possible by the planar surface with gradients of substrate cues deposited using microfluidic devices specifically designed. In this way, conventional photolithographic methods can produce the devices realizing 2D and 3D designed brain-on-chip models. Nevertheless, as the subject relating to fabrication of both 2D and 3D design chips, it is highlighted that the use of photolithographic procedure, which is multistep and needs a reasonable time, cannot be avoided, since 3D structural molds are necessary for stamping and transferring in either case.

On the other hand, technologies based on soft lithographic procedures above-mentioned have been widely used, while microstructures formed by self-organized phenomena of polymer materials have been applied to biological research^19–22^. Formation of various surface patterns, such as wrinkle, line and crack, was achieved by means of thin film deposition^23^, plasma oxidation^24^, and ultraviolet/ozone radiation^25^, and moreover the researchers explained the forming mechanism in a structure of the stiff surface layer on a soft material^19,23^. Our group adopted the deposition of a diamond-like carbon (DLC) thin film, one of hard coatings, on the surface of PDMS, and has created some microstructures and their patterns to apply to cellular scaffolds^26–30^. Here, DLC thin films^31^, which consist of mainly carbon and hydrogen, present the biocompatible, excellent tribological and chemically inert properties, and have been widely applied to medical uses as biomedical components^32,33^. Also, PDMS is an elastomer featured by its formability, transparency, biocompatibility and oxygen permeability properties. Through our recent research, the followings have been found. Firstly, when the DLC thin film was deposited onto a PDMS substrate directionally stretched in advance, a wrinkle microstructure with a periodically regular linear pattern was formed on its surface^26,28^. Cross-sectionally a wave-like concavo-convex shape was created at the size of about 3–12 μm in width and about 1–3 μm in height. The morphologies and sizes of the microstructures were controllable within a certain range by the deposition conditions. Next, when cultured on the microstructural surfaces of DLC deposited PDMS substrates, mouse fibroblast and myoblast cells adhered more selectively on the surfaces, and tended to spread along the linear patterns^27,29^. Moreover, using the design patterns fabricated by partially depositing DLC thin films it was demonstrated that human mesenchymal stem cells (hMSCs) selectively attached on the DLC deposited wrinkle areas, and as bases around the areas the pseudopodia extended toward neighbor wrinkle areas^30^. In the meanwhile, as other research on combination of DLC and PDMS, especially in terms of the biological applications, usages of PDMS substrates formed 3D structures in advance, such as micro-scale pillar patterns^34^ and grooved surface^35^, were reported. In addition, as polymers other than PDMS, research to enhance the use of polyethylene for various medical applications by DLC coatings have progressed, in which the DLC thin films were improved by incorporation of fluorine^36^ and silicon^37,38^, and laser-based processing techniques^38^. It is notable that the research including our studies used the plasma chemical vapor deposition (CVD) method with hydrocarbon gases for DLC coatings, because the deposited hydrogenated carbon films were more tightly attached to polymeric materials compared to more rigid amorphous carbon films fabricated by the other methods.

In this manner, our research indicated that the DLC deposited PDMS substrates have the ability to be adhered cells more tightly and changed the cell shapes by designing the adequate patterns composed of the deposited areas. In this study, we applied these functions to effective control of neuronal cells, positioning somata and guiding axons, toward the development of brain-on-chip models. And we were intended to create no guiding groove plane-type substrates, on which plenty of independent axons were taut in a straight line, for evaluation relating to axon behaviors in vitro. In addition, this study aimed to produce an uncomplicated method with only DLC thin film deposition. The method allows extrication from dependence on conventional soft lithographic technologies requiring multiple stages and their treating times, and greater flexibility for the observation and various evaluations of neuronal cells cultured, because a top cover essential to driving of microfluidic devices is unnecessary for the method proposed in the study.

## Materials and methods

### DLC/PDMS substrate fabrication

**Figure 1:**
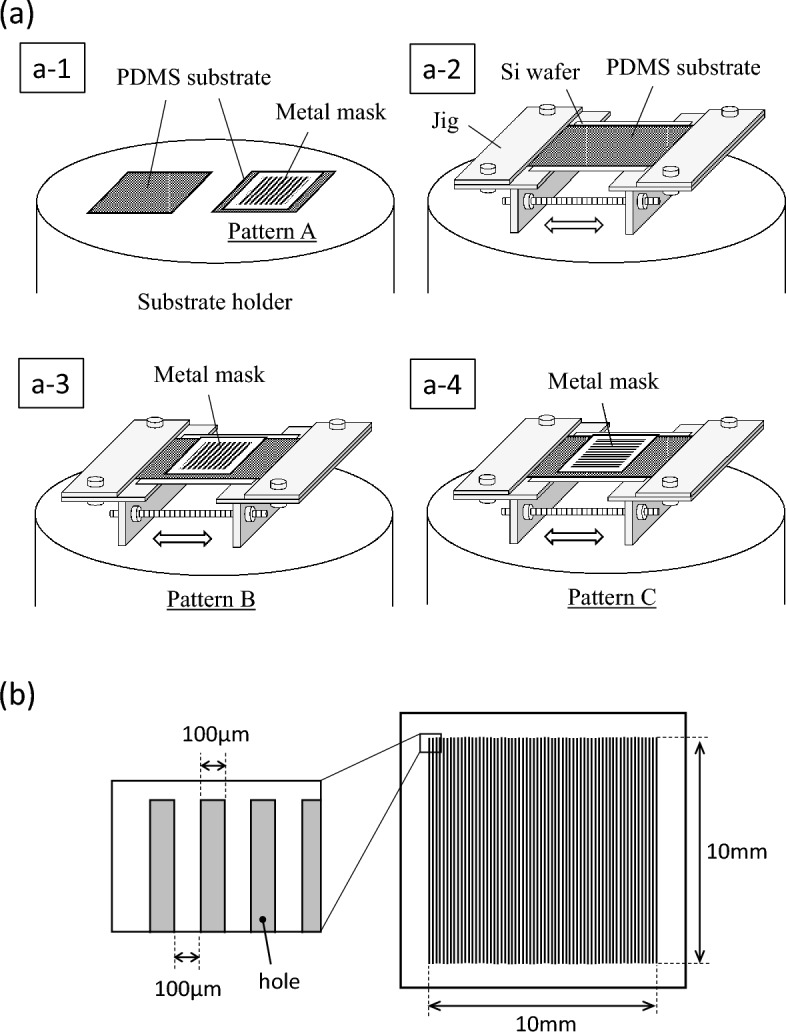
Schematic overview of how to place substrates during DLC thin film deposition: (**a**) a PDMS substrate on the substrate holder with or without a jig and (**b**) the design of metal mask covered on the PDMS substrate.

DLC thin films were deposited on PDMS substrates without an interlayer by means of a plasma chemical vaper deposition (CVD) using methane gas. The depositions were performed using a vacuum chamber equipped with an inductively-coupled plasma (ICP) source on the top^26,27^. The ICP source can create a down-flow plasma toward the substrate holder applied a negative bias voltage with a RF generator. An elastomer material, PDMS, was used as the substrates, and the flat plates (thickness: approximately 1 mm) were made by mixing liquid prepolymer with curing agent (SILPOT 184 W/C: Dow Corning Toray Co., Ltd.) at a ratio of 10:1. The curing was carried out by still standing at room temperature for about 2 days. The DLC deposited PDMS (DLC/PDMS) substrates fabricated in the study were grouped by the presence or absence of the use of a metal mask during the depositions. In the case without the mask, called hereafter “whole surface deposition (Test A)”, the flat PDMS substrates were put directly (the left of a-1 in Fig. 1a) or with fixed in a jig (a-2 in Fig. 1a) on the substrate holder. The jig was used for fastening and stretching the PDMS substrate in one direction as can be seen in the figure, and the tensile strain was set at 0.05 and 0.2. Also, the deposition time was set at 2 and 6 min. These varying ranges were determined based on the results of our previous study^26^, which clarified that more ordered structure was formed only under the limited conditions in our CVD system, that is, the tensile strain of 0.05 to 0.2, and the deposition time of 2 to below 10 min. The sample with no strain (put directly on) was prepared for evaluating larger point of difference in the deposited surfaces by the presence or absence of the strain. A silicon wafer was served as a physical and electroconductive support. The other case using the mask, called hereafter “patterning deposition (Test B)”, was carried out using the PDMS substrates covered with the mask, which had 50 straight holes (100 μm width and 10 mm length) with each interval of 100 μm width, illustrated in Fig. 1b. Here, DLC thin films were deposited at three different settings, Pattern A, B and C. For Pattern A, the PDMS substrates covered with the mask were directly put on the substrate holder as shown in the right of a-1 in Fig. 1a. The illustrations of a-3 and a-4 in Fig. 1a present the settings of Pattern B and C, respectively, indicating that the metal mask covered the PDMS substrates stretched at the tensile strain of 0.2 in one direction using the jig just like a-2, while the longer direction of the holes of the mask was different, namely, perpendicular and parallel to the tensile direction at Pattern B and C, respectively. The patterning deposition was all performed at the deposition time of 2 min. After the depositions, the mask was carefully removed, and the strain was slowly unloaded. In both whole surface deposition (Test A) and patterning deposition (Test B), the other deposition conditions were same, the substrate bias voltage of − 650 V and the pressure of about 0.8 Pa, and argon plasma irradiation of 90 s was carried out at the bias voltage of − 400 V for cleaning before the depositions. The thicknesses of the DLC thin films under above deposition conditions were estimated to be about 14 and 42 nm, respectively, at the deposition times of 2 and 6 min from the deposition rate, 7 nm/min, which was obtained by thickness measurements when DLC thin films were deposited on silicon wafers for a long time^26^.

Raman spectroscopy analysis was carried out using an argon laser of 541 nm with the conditions: laser power of 5 mW, exposure time of 7 s and cumulative number of 5, for the surfaces of the DLC/PDMS substrates. The microstructures of the DLC/PDMS substrate surfaces were observed using a confocal laser microscope (CLM) (VK-9700, KEYENCE Co.).

### Culture of neuronal cells

Human neuroblastoma cells, SH-SY5Y (ECACC General Collection, Public Health England) purchased from KAC Co. (Japan) were used for this study. The cells were cultured with SH-SY5Y medium (F12/Eagle’s Minimum Essential Medium supplemented with NEAA and 15% fetal bovine serum) (KAC Co. (Japan)) at 37 °C in a 5% CO_2_ incubator. After confluency was attained in 2–3 day culture including one or two passages, the cells at passage 16 or 17 were used to prepare the cell suspension with a cell concentration of 4.5 × 10^4^–6.0 × 10^4^ cells/ml.

The culture tests using the DLC/PDMS substrates fabricated were carried out according to the following procedure. The substrates were immersed into ethyl alcohol (99.5%) for a few seconds for sterilization, phosphate-buffered saline (PBS) for rinsing, followed by collagen solution (0.01%) for cell adhesion. The substrates were put in wells of a 24 (for Test A) or 6 (for Test B) well plate, and the SH-SY5Y medium followed by the cell suspension were dispensed in them, being a seeding density of 1.4 × 10^4^ (Test A) or 4.7 × 10^3^ (Test B) cells/cm^2^. After culturing within 48 h, the medium was replaced with Neurobasal medium (Invitrogen) containing 2% B-27 supplement (Invitrogen), 0.5 mM GlutaMAX (Invitrogen) and 10 μM all-trans-retinoic acid (ATRA) (FUJIFILM Wako Pure Chemical Co.) to promote differentiation into neuronal phenotype, and the cells were grown up to 6 (Test A) to 5 (Test B) days at 37 ℃ in a 5% CO_2_ incubator, refreshing the medium every 2–3 days^39^.

### Cell staining evaluation

Immunofluorescent staining evaluation was performed with the following standard procedure. After removing the mediums, the cells were washed three times with PBS. The cells were fixed in 4% formaldehyde solution in PBS for 30 min, and permeabilized with 0.1% Triton X-100 in PBS at room temperature for 15 min, followed by blocked with 5% normal goat serum (FUJIFILM Wako Pure Chemical Corp.) in PBS at room temperature for 90 min. The cells were incubated with the primary antibodies at 4℃ overnight, and then incubated with the secondary antibodies at room temperature for 2 h. As the primary and secondary antibodies, mouse anti-Tuj1 (neuronal class III β-tubulin) antibody (R&D Systems, MAB1195) diluted 1:2000 in 1% normal goat serum PBS, and Alexa Fluor 546 goat anti-mouse antibody (Invitrogen, A-11003) at 1:1500 in PBS were used, respectively. DAPI (PromoKine) was used for staining the nucleus. As a negative control, cells on cover glasses without the primary antibody and with the secondary antibody were prepared. The stained cells were observed by a fluorescence microscope, and as for Teas A, representative five separate regions of each substrate were photographed. In the case of Test B, the entire region where the DLC thin films were deposited was photographed, and the experiment was carried out in triplicate.

## Results and discussion

### Structural evaluation of DLC thin films

**Figure 2:**
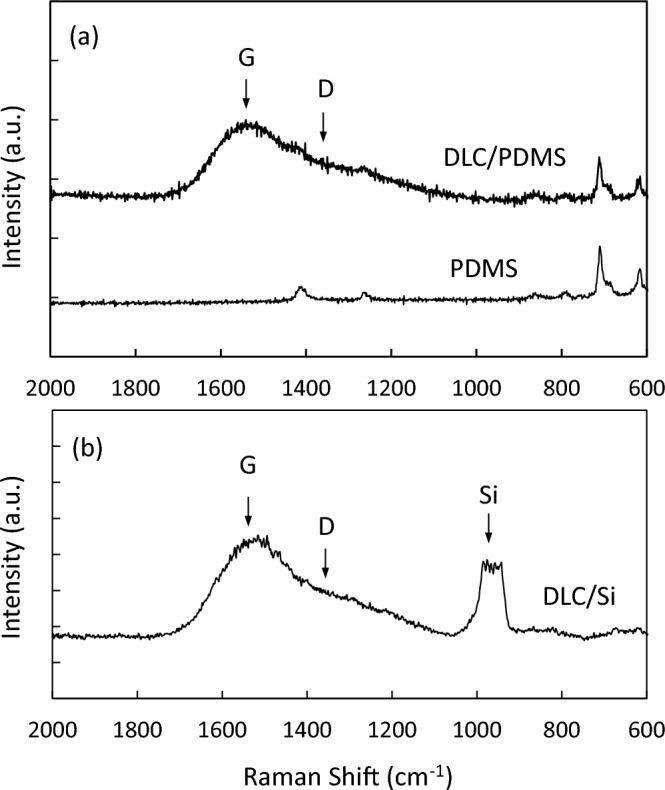
Raman spectra obtained from (**a**) the DLC thin film deposited PDMS (DLC/PDMS) substrate at the tensile strain of 0.2 and the deposition time of 2 min, and a PDMS substrate (PDMS), and (**b**) the DLC thin film deposited silicon substrate (DLC/Si).

Figure 2a shows Raman spectra obtained from the DLC/PDMS substrate fabricated at the tensile strain of 0.2 and the deposition time of 2 min in Test A, and a PDMS substrate (PDMS) as reference. Additionally, the spectrum in Fig. 2b represents the Raman shift of a DLC thin film deposited on not PDMS but a silicon wafer often used as the conventional substrate for the Raman analysis. Both deposited thin films indicated typical Raman spectra corresponding to so-called diamond-like carbon structure consisting of graphitic (G) and disorder (D) peaks (around 1540 and 1350 cm^−1^, respectively) in conjunction with peaks attributed to PDMS (615, 710, 780, 850, 1250 and 1405 cm^−1^) or silicon (around 950 cm^−1^). Decomposing the spectra into the D and G peaks using a Gaussian function, the intensity ratios of D to G peak, ID/IG, and G and D peak shifts of DLC/PDMS and DLC/Si were calculated to be 0.447 and 0.457, 1545 and 1540 cm^−1^, and 1350 and 1350 cm^−1^, respectively. Namely, it is noted that both DLC thin films on PDMS and silicon presented almost same ID/IG, G and D peak shifts. Raman spectra of DLC thin films are often explained according to the interpretation that ID/IG and G peak shift reflect a size of *sp*^2^-bonded clusters and a concentration of *sp*^3^ sites, respectively^40^. Considering the Raman results stated above, it can be estimated that the DLC thin films deposited on PDMS in this study have a structure comparable to those on a silicon wafer used conventionally. Nevertheless, the more accurate comparison must be done taking the effect of residual compressive stress on the G peak shift into account^41^. The magnitude of the stress-induced shift to higher wave number of G peak was reported as − 4.9, − 4.1 and − 1.9 cm^−1^/GPa for a disordered graphite, a tetrahedral amorphous carbon (ta-C) and an amorphous carbon (a-C), respectively^41,42^. However, at this time, the effect cannot be estimated, because G peak positions in a stress-released state of the DLC thin films on PDMS and silicon in the study have not been obtained. More definitely the compressive stresses actually arisen in the films must be measured.

### Whole surface deposition (Test A)

**Figure 3:**
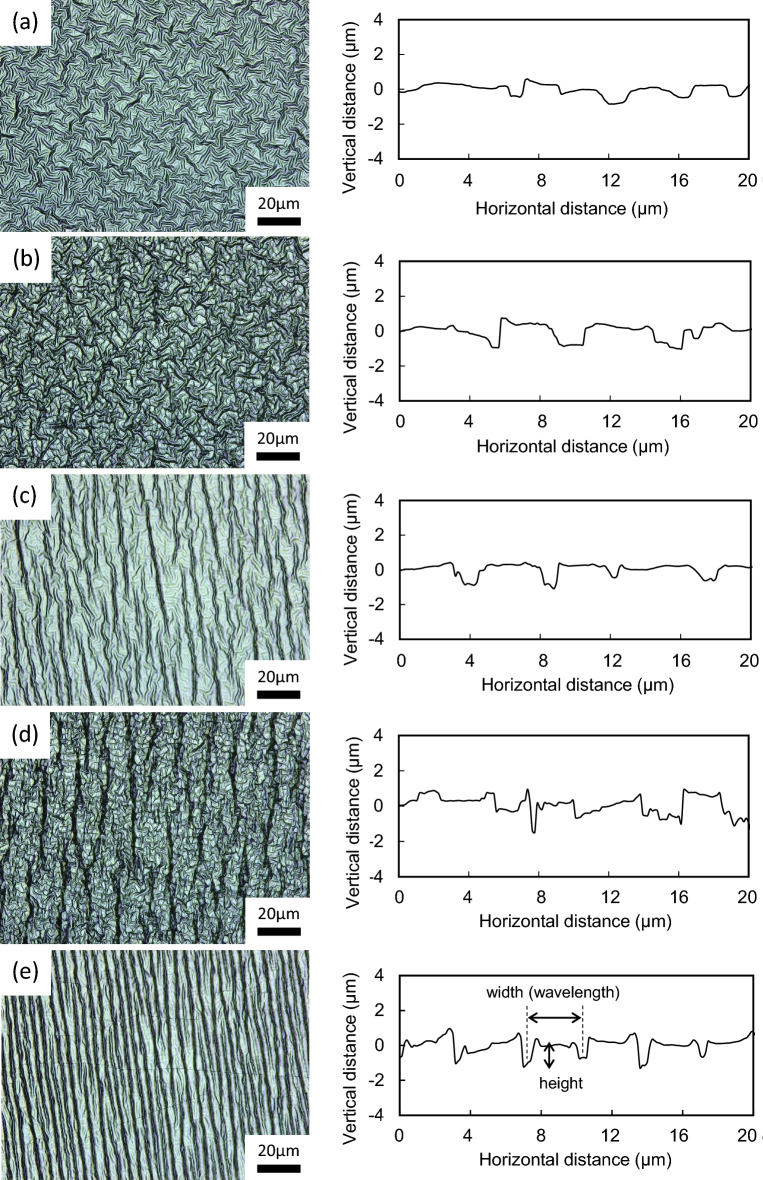
The typical surface images observed by CLM as for DLC/PDMS substrates fabricated at the tensile strains/the deposition times of (**a**) 0/2, (**b**) 0.05/6, (**c**) 0.05/2, (**d**) 0.2/6 and (**e**) 0.2/2 min. The tensile strain was applied from side to side in the images. The right side represents the cross-sectional profile measured at each condition of (**a**)–(**e**).

The typical surface images observed by CLM are shown in Fig. 3 as for the DLC/PDMS substrates fabricated at the tensile strains/the deposition times of (a) 0/2, (b) 0.05/6, (c) 0.05/2, (d) 0.2/6 and (e) 0.2/2 min. In the figure, the right side represents the cross-sectional profile measured at each condition of (a) to (e). In the case of the tensile strains of 0.05 and 0.2, the PDMS substrates were stretched from side to side in the images. It is noted that the surface morphologies strongly depended on the conditions, and the feature can be categorized into two groups, (a, b) and (c–e), in terms of the orientation of the wrinkles. At the tensile strains/the deposition times of (c) 0.05/2, (d) 0.2/6 and (e) 0.2/2 min, as one group, the wrinkles oriented perpendicularly to the direction of strain can be seen in varying degrees. More periodic wrinkles appeared at (e) 0.2/2 min, and as the average width (wavelength) and height of the wrinkles, about 3.0 and 1.2 μm were measured by the cross-sectional profile (in detail, Fig. S1). Adding a little more detail, the cross-sectional profile indicates that the wrinkles became a distinguishing shape, that is, not ordinary sinusoid but more like period-doubling. The wrinkles observed at (c) 0.05/2 and (d) 0.2/6 min showed a weaker orientation, as can be seen in the cross-sectional profiles. At the other group, disordered wrinkles without clear orientation were observed at (a) 0/2 and (b) 0.05/6 min.

The surface morphology can be explained by a model that wrinkles in the combination of a thin, stiff coating film and a thick, soft substrate are formed as buckling of the film caused by a compressive stress arisen from the considerable mismatch of both elastic moduli^23–25^. In this study, at the condition of no strain the compressive stress was brought by thermal expansion and shrinkage of PDMS during the deposition process. While, at the conditions with PDMS stretched the compressive stress was provided by not only the thermal expansion and shrinkage but also the stretching in advance and removal. These compressive stresses were roughly calculated in Fig. S1. In either case, the strong difference in elastic moduli between the DLC thin film and PDMS led to the wrinkle formation. Therefore, using the equation given by the above model, the width and height of wrinkles were estimated to be 1.8–2.3 and 0.80–1.0 μm, respectively, in the case of (e) 0.2/2 min, given the DLC thin film thickness of 14 nm (in detail, Fig. S1). It was found that the size of wrinkles formed in the study was somewhat larger than that calculated by the model previously proposed. The result suggests a possibility that a thermally-modified layer at the top surface of the PDMS during argon plasma cleaning before the DLC deposition contributed to formation of larger size than predicted. The two processes also might induce the periodic-doubling profile consisting of smaller and larger waves. That is, it is implied that firstly thermal expansion and shrinkage of PDMS surface during argon plasma irradiation made the smaller waves, and then by the DLC deposition the larger waves were created with the smaller ones included. On the other hand, in the case of no strain, it can be understood that a factor that wrinkles should be formed with the regularity cannot be found and the wrinkles were randomly oriented. However, even though the strain was applied, the condition of 0.05/6 min resulted in little regularity. Our previous study indicated that as the deposition time increased, the formed wrinkles tended to be irregular^26^, and the phenomenon reappeared at the condition of 0.05/6 min of this study. It is highly probable that the irregularity was brought due to excess thermal elevation of PDMS at a longer deposition time, and that became more pronounced when the strain was smaller. Also, it is guessed that since restrained condition of PDMS by the jig disturbed the free thermal expansion to some extent, the wrinkles were not created with the regularity unlike the model assuming the free expansion.

**Figure 4:**
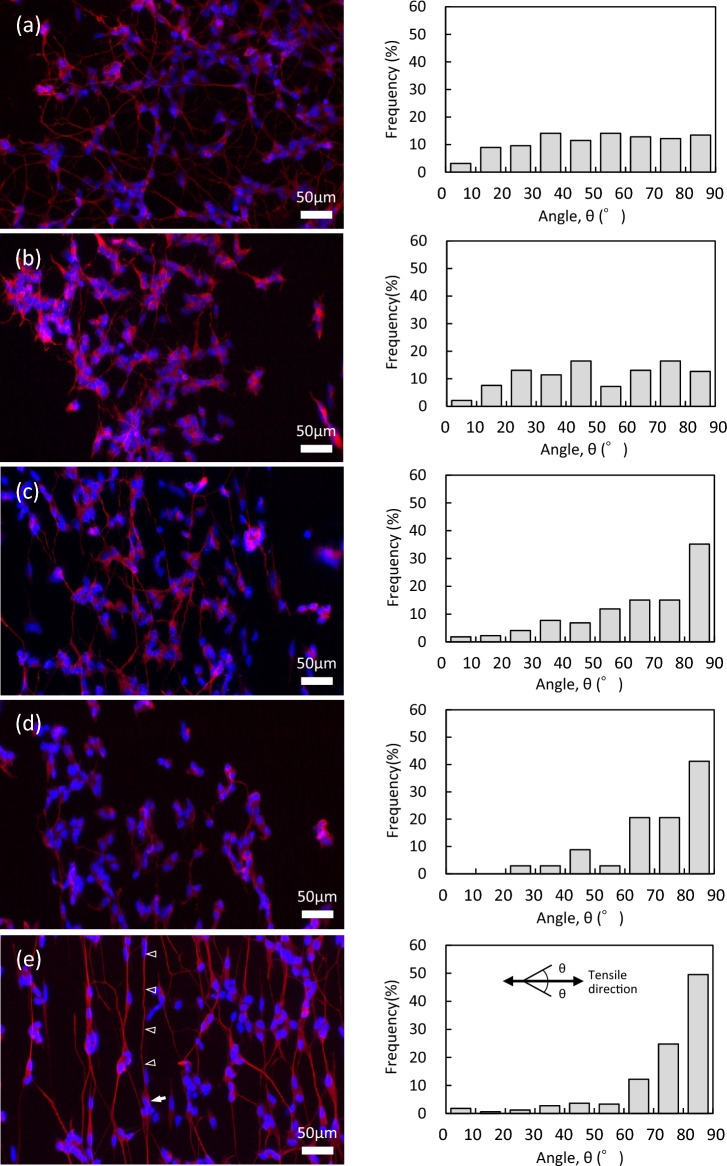
The typical images of cells observed by a fluorescence microscope as for DLC/PDMS substrates fabricated at the tensile strains/the deposition times of (**a**) 0/2, (**b**) 0.05/6, (**c**) 0.05/2, (**d**) 0.2/6 and (**e**) 0.2/2 min. Cell nuclei stained in blue with DAPI and stained red represents the expression of Tuj1. The cell indicated by an arrow presents the fine straight axon with more than 200 μm in length (marked by triangles). The right side represents the histogram relating to the distribution of outgrowth directions of axons obtained from each condition of (**a**)–(**e**). The direction was measured as the angle, θ (the tensile direction = 0° and the perpendicular direction = 90°).

The typical images of cells (6 days in culture) observed by a fluorescence microscope are shown in Fig. 4a–e, concerning the DLC/PDMS substrates fabricated at same conditions as Fig. 3a–e. In the images, cell nuclei were stained in blue with DAPI and red obtained by immunofluorescence staining represented the expression of Tuj1, which eccentrically located in mainly cell bodies and axons. Focusing on the axons, the thicker and stronger outgrowths were observed at cells on the substrate of (e) 0.2/2 min, and in the cases of other substrates thinner, weaker and shorter ones were seen. In addition, it can be found that the connection between cells by axons developed more at (a) 0/2 and (e) 0.2/2 min. Using the fluorescence microscope images, taken an example in Fig. 4a–e, the outgrowth directions of axons were investigated by extracting 70–300 cells for each condition, and the distributions are displayed at the right side of Fig. 4 as the histogram. The direction of each cell was measured as the angle, θ, when the tensile direction (from side to side in the figure) and the vertical direction were 0° and 90°, respectively. The figure obviously indicates that for the substrate fabricated at 0.2/2 min, about 50% of cells stretched the axons in the angles of 80°–90° (about 90%, in 60°–90°), that is, the direction along regular wrinkle pattern formed perpendicularly to the direction of tensile strain, as shown in Fig. 3. As against that, the cells cultured on the substrates with irregular patterns of (a) 0/2 and (b) 0.05/6 min, showed omnidirectional stretching of axons. In addition, as for (c) 0.05/2 min and (d) 0.2/6 min, being some ordered wrinkle patterns observed (see Fig. 3), the axons had the tendency to outspread in the direction of higher angles. These results demonstrate that the pattern of wrinkles formed on the substrate served effectively to decide and guide the stretching direction of axon outgrowth. It has been ever reported that the microstructures with moderate sizes affected cellular behaviors, and in particular, a straight pattern allowed cells to be stretched linearly along it due to filopodium extension^20,29,43^. This study clarified that when neuronal cells were cultured on wrinkle microstructure in a linear fashion, even the axons extended along the pattern. The cell indicated by an arrow in Fig. 4e presents the fine straight axon with more than 200 μm in length (marked by triangles).

### Patterning deposition (Test B)

**Figure 5:**
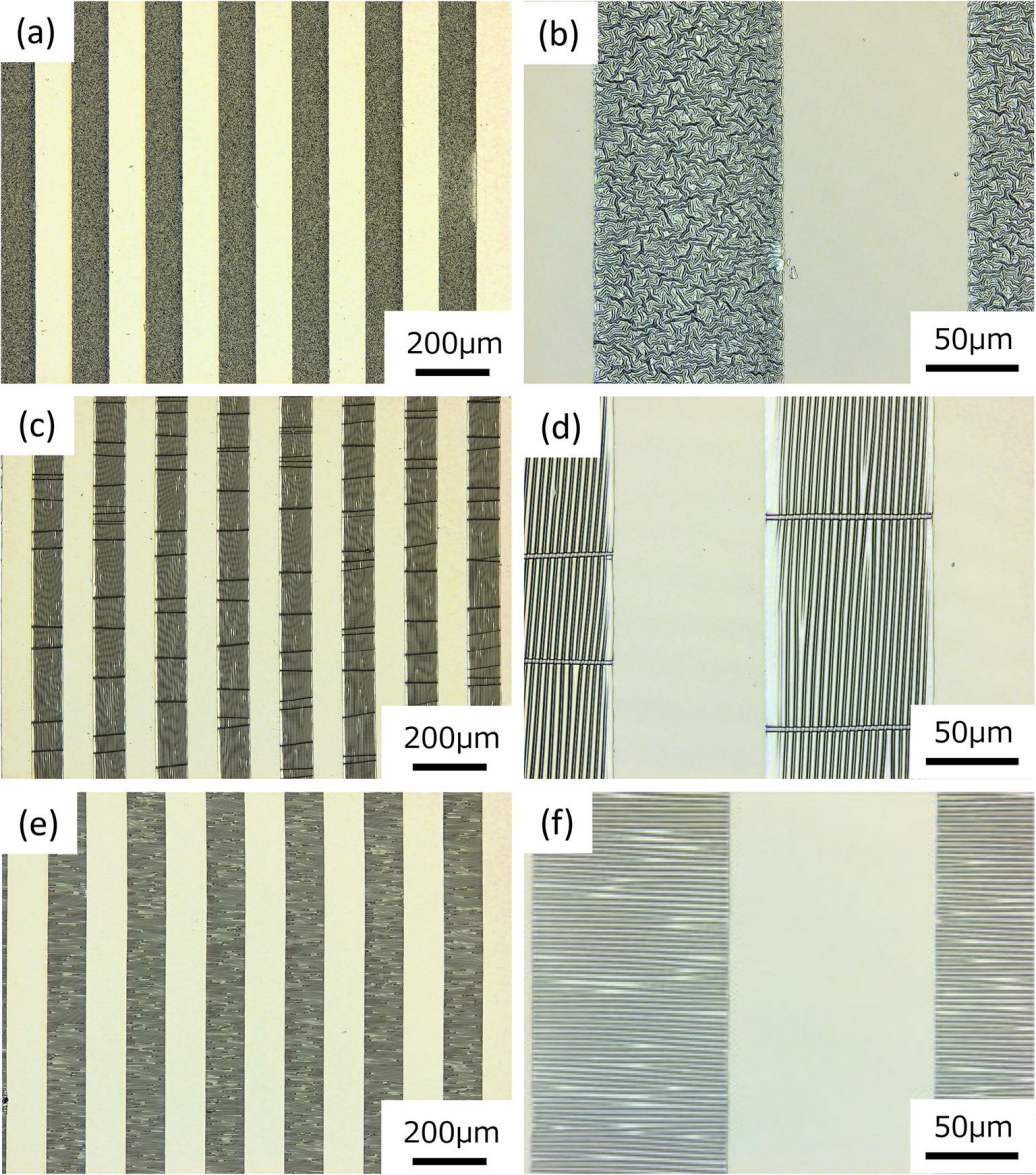
The typical observation images by CLM as for (**a**, **b**) Pattern A, (**c**, **d**) B and (**e**, **f**) C. (**b**), (**d**) and (**f**) are the magnifications of (**a**), (**c**) and (**e**), respectively. The tensile strain of 0.2 was applied from side to side for (**c**, **d**) Pattern B and from up to down for (**e**, **f**) Pattern C.

The typical observation results obtained by CLM are shown in Fig. 5 as for Pattern A (a, b), B (c, d) and C (e, f), and (b), (d) and (f) are the magnifications of (a), (c) and (e), respectively. The tensile strain of 0.2 was applied from side to side and from up to down in the figure for Pattern B and C, respectively. As for all patterns, the DLC thin films were deposited on a multiple of rectangular areas where the holes were made in the used metal mask, and various wrinkle patterns were formed on the areas. However, the patterns were largely different depending on the conditions adapted, resulting in disordered (irregular) wrinkles and ordered (regular) wrinkles in a linear manner at Pattern A, and B and C, respectively. Also, the directions of lines of B and C were parallel and perpendicular, respectively, to the longer direction of the DLC deposited rectangular areas. Meanwhile, note that the directions of lines of B and C were both perpendicular to the stretching direction of the PDMS substrates. The data obtained from CLM indicated that the widths/heights of the linear wrinkle patterns of B and C were approximately 5.1/1.3 and 3.2/0.7 μm, respectively (see Fig. S2).

**Figure 6:**
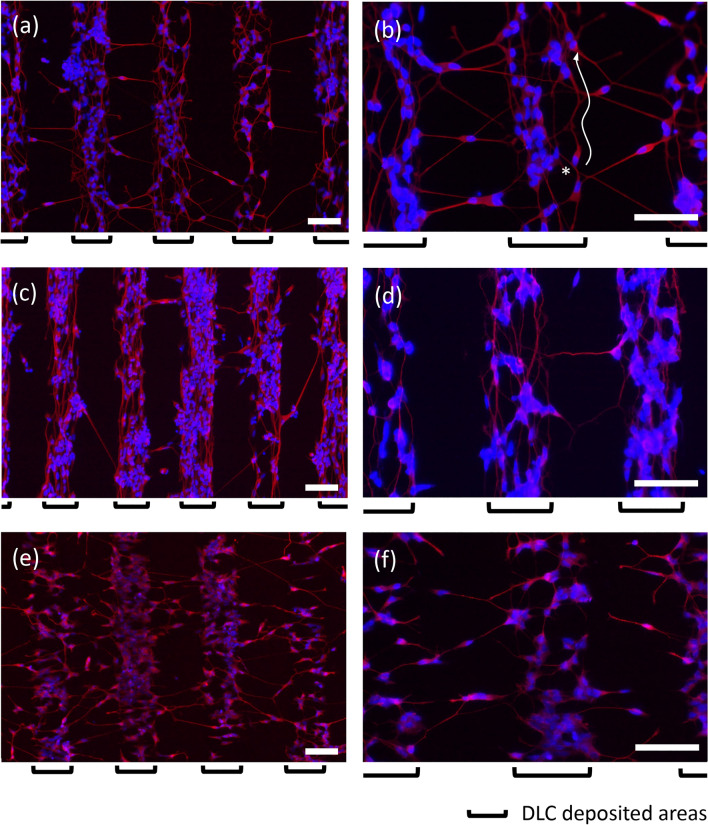
The fluorescent microscope images obtained from the substrates of (**a**, **b**) Pattern A, (**c**, **d**) B and (**e**, **f**) C. Axons and cell bodies stained in red were merged with the nuclei in blue. The cell marked with an asterisk expanded the axon without any departure from the DLC deposited areas through meandering. Scale bar corresponds to 100 μm.

The fluorescent microscope images in Fig. 6 demonstrate the results obtained from the substrates of Pattern A (a, b), B (c, d) and C (e, f). Here, axons and cell bodies stained in red by Tuj1 expression can be also seen merged with the nuclei in blue. The cell bodies including the nuclei were almost attached on the DLC deposited areas for any substrates. In regard to this point, only nucleus-stained images (Fig. S3) comparing Pattern A with the case of no strain of Test A (without the metal mask) can be illustrative. Meanwhile, each pattern formed by stretching of axons was quite different for the substrates of Pattern A, B and C. The most notable characteristics of the patterns can be found in the stretching directions of the axons on the DLC deposited areas. That is, the directions in Pattern B and C were relatively parallel and perpendicular, respectively, to the longer direction of the deposited rectangular areas. When compared Fig. 6 with Fig. 5, it can be noted that the directions were same as those of lines formed by wrinkle patterns, implying that even in the case of Test B, the axons grew in accordance with the patterns established on the areas. Interestingly, it was found that the axons extended to the areas without DLC thin films for any substrates, and it followed that the axons connected the cell populations on the DLC deposited areas to each other. The interconnecting axon is more notable feature in the characteristic structures created in the study, and the qualitative and quantitative results will be discussed hereafter. The whole structure resembles the shape of ladder lottery called ‘Amidakuji,’ which is one of the popular methods used for decision in Asia.

**Figure 7:**
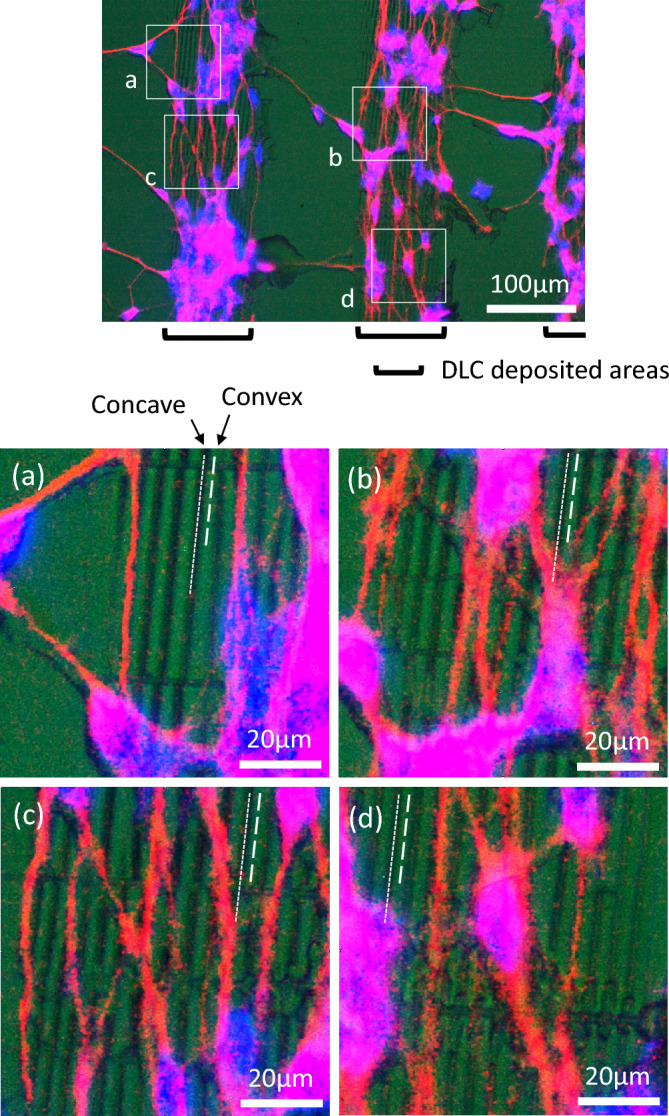
As for Pattern B, images obtained by merging the fluorescent image of nuclei in blue and Tuj1 in red, and the optical image (green split in three primary colors) of the substrate surface, taken at the same position. The lower images (**a**–**d**) are the magnifications of four areas in the upper image.

Figure 7 was obtained as for Pattern B by merging the fluorescent image of nuclei in blue and Tuj1 in red, and the optical image of the substrate surface which was taken at the same position, together. The optical image was split in three primary colors, and the green image was used to distinguish cells represented in the other colors. The magnifications of four areas in the upper image were shown in the lower images, (a–d), respectively. As can be seen in Fig. 5d, the ordered wrinkle structure in a linear fashion can be confirmed by contrasting density of green color on the DLC deposited areas, that is, the lines of deeper green color correspond to the concave shape of the wrinkle. It is relatively clearly recognized that most axons stained in red were growing at the bottom of concaves. A number of studies have examined the effects of various surface topographies on the behaviors of cells, mainly fibroblasts, epithelial cells and mesenchymal stem cells (MSCs). Fujita et al. reported the dynamic behaviors of living MSCs on nanogrooved patterns by time-lapse observation of filopodia, presenting cell alignment along the grooves and the mechanism model^43^. The model proposed that a filopodia extends over the ridge line capable to form wider focal adhesions and rapidly retracts in the direction perpendicular to the line due to fragmentated focal adhesions. The phenomenon observed at the neuronal cells, stretching at the bottom of concaves, seems to be different from the case of MSCs, because MSCs have a larger size than SH-SY5Y and also the size of the nanogrooves is smaller than that of the wrinkle in the study. That is, here, the fact that the formed wrinkle structure had the comparably sized concaves to axons should be emphasized, and it is highly probable that ease of fitting with concaves became one of important factors for guiding axons. In fact, it is noted from Fig. S2a that the width of the concaves was estimated to be about 2 μm, which is comparable in size to finer axons. It was demonstrated that the concaves operated similarly to guiding grooves fabricated by conventional photolithographic methods.

**Figure 8:**
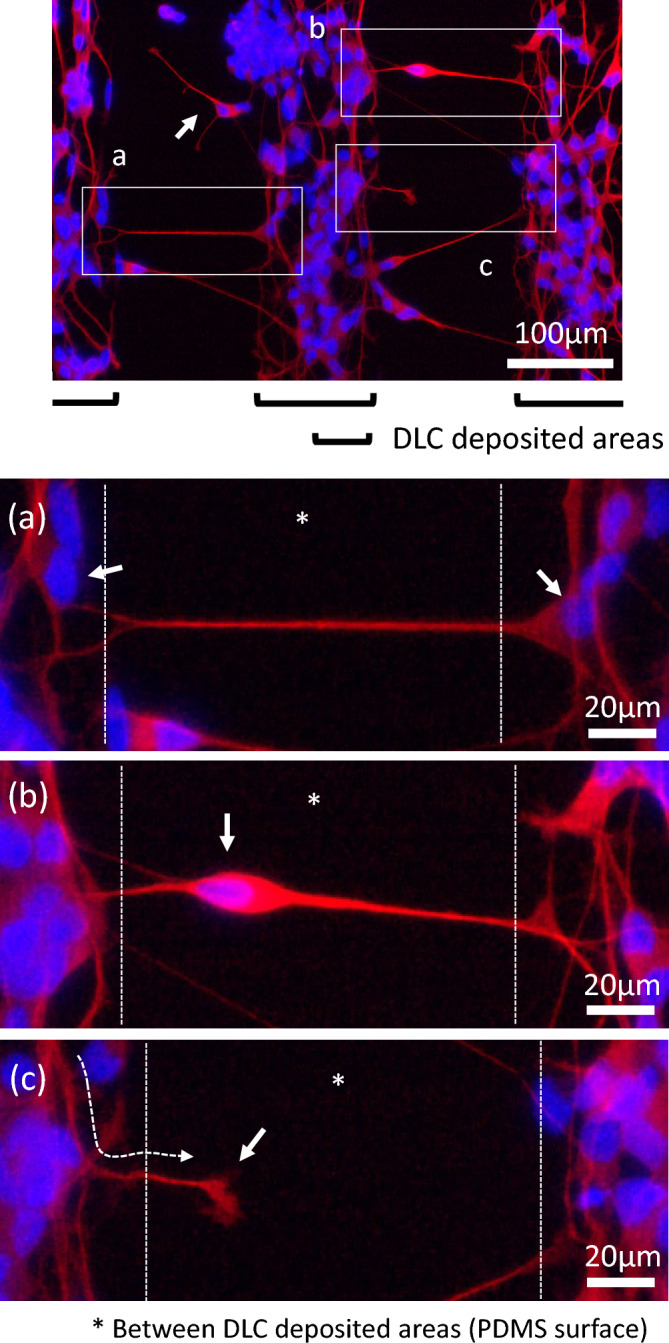
The fluorescent microscope images obtained from Pattern B. Axons and cell bodies stained in red were merged with the nuclei in blue. The lower images (**a**–**c**) are the magnifications of three areas in the upper image.

In Fig. 8, concerning Pattern B, the axons on PDMS surface without wrinkle patterns between DLC deposited areas are presented in more detail: the lower images, (a–c), are the magnifications of three areas of the upper image. These lower images demonstrate how the axons behaved as main three patterns. The first one shown in Fig. 8a is the most frequent pattern, and the axon stretching from the cell (pointing the nucleus by the arrow) on the right DLC deposited area reached the left one to connect to the cell (probably pointed by the arrow) on the left area. It is noted that the axon formed a remarkably straight shape like a taut rope, and was likely to approach by the most direct way. The case of Fig. 8b also appeared more often, indicating that one cell body existed on the PDMS surface between the DLC deposited areas, and mediated the connection. The case that the axon growth was in the middle of reaching the other side, as in Fig. 8c, was also occasionally observed, and in this instance the axon extended downward, i.e., the direction of the linear wrinkle pattern, from the upper left corner to veer away to the right of the image, as guided with the dotted line arrow. It is highly probable that the nerve growth cone, pointed by the arrow, was formed at the end of the elongating axon. The cell with the arrow in the upper image of Fig. 8 also had the axon not reaching to the other side while more growing compared to that in Fig. 8c. It can be guessed that when these cells cultured longer, the growth cone will get to the area with DLC thin film deposited, resulting in the connection between the respective cells existed on adjacent different DLC deposited areas, as with the case of Fig. 8a.

To make clearer comparison among Pattern A, B and C, the quantitative treatments of the fluorescent microscope images were carried out. The images in Fig. 6 present the typical examples, but meanwhile, the photographs were taken throughout the entire range of each substrate. Here, the direction of axonal elongation on a substrate, θ, is defined as shown in Fig. S4, which is the schematic diagram of the DLC/PDMS substrate.

**Figure 9:**
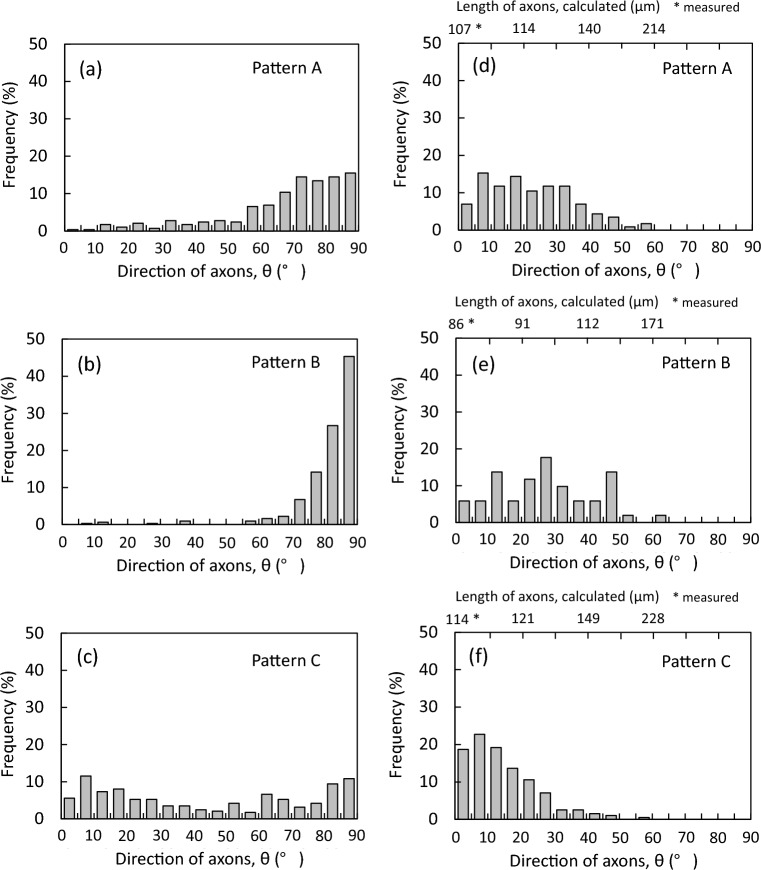
As for the DLC deposited areas (# in Fig. S4), the histogram of the direction of axons, θ, of (**a**) Pattern A, (**b**) B and (**c**) C. As for the intervals between the DLC deposited areas (* in Fig. S4), the histogram of the direction of axons, θ, of (**d**) Pattern A, (**e**) B and (**f**) C.

At first, as for the DLC deposited areas, Fig. 9a–c shows the histogram of the direction of axons, θ, obtained using respective about 290–310 cells extracted from the images of Pattern A (a), B (b) and C (c) (see Fig. S5). The figure indicates that the cells on Pattern B clearly exhibited a tendency to extend the axons toward the perpendicular direction, namely, the longer direction of the deposited areas. This means that the growth of axons can be certainly guided by the wrinkle structure, since the direction of the formed straight wrinkle coincided with the longer direction. On the other hand, the histogram of Pattern A implies that the axons were more likely to stretch in a vertical direction despite the disordered wrinkle pattern unlike Pattern B (see Fig. 5). The phenomenon in this manner is guessed to be caused by the shape of DLC deposited areas. That is to say, that means that axons have little choice but to grow toward the longer direction along the shape, because the axons prefer the inside of the DLC deposited areas and dislike going into the outside. As can be seen in Fig. 6b, the cell marked with an asterisk expanded the axon without any departure from the DLC deposited area through meandering. The phenomenon can be confirmed even in the case of Pattern C, which has the wrinkle pattern of the direction perpendicular to the longer direction of the DLC deposited rectangular areas. For that, concerning Pattern C, not only the direction of lower angles but also the direction of higher angles were chiefly observed. That is, it can be recognized that the growth direction of axons was not only controlled by the microstructure but also governed indirectly from the shape of the DLC deposited areas. This strongly suggests that Pattern B having the design in which the straight wrinkle lengthens in a longitudinal direction of the deposited areas allows more axons to stretch in one direction by synergic effect.

Next, as for the stretching axons on the intervals between the DLC deposited areas, the direction is shown as the histogram in Fig. 9 (Pattern A (d), B (e) and C (f)). Respective about 50 to 230 cells were extracted from the images for preparation (see Fig. S6). The larger trend observed from three histograms may be the difference in the position of increase in frequency, noted that the positions changed relatively from lower angles of Pattern C (0°–15°) through middle angles of Pattern A (5°–20°) to higher angles of Pattern B (10°–50°). It is very likely that the stretching direction of axons on the intervals is susceptible to indirectly the direction of wrinkle patterns and directly the primary growing direction of axons extended from the cell. The wrinkle pattern of Pattern C is a linear shape perpendicular to the longer direction of the deposited areas, namely the direction of θ = 0°, toward which the cells tended to extend axons (see Fig. 9c). It is suggested that the axon outgrowth began toward the direction of lower angels near θ = 0° and proceeded on the intervals with the direction keeping, as observed in Fig. 6e,f. Nest, seeing the histogram of Pattern B (Fig. 9e), it is true that the frequencies of lower angles are relatively low, meanwhile the higher angles are observed more. Based on acknowledgment of the phenomenon in Pattern C, this may be interpreted as follows. As above mentioned many times, Pattern B has the wrinkle pattern of a linear shape parallel to the longer direction, the direction of θ = 90°. When the axons of cells on the deposited area traveled to the neighboring deposited area, their stretching directions on the intervals depended strongly on their own growing directions on the deposited area, and consequently the axons extended in the higher angles rather than lower angles near θ = 0°. Pattern A is a disordered pattern, and has the wrinkles of not specific directions, perpendicular and parallel, but every direction in a way. For that, the axons were not subject to the limitation of growth to the specific directions, and the position of increase in frequency was in the range of neither lower angles nor higher angles to become the range intermediate between Pattern B and C. Moreover, the length of axons connecting between the DLC deposited areas was calculated from the width of intervals and the direction of axons to be added at the upper ends of histograms of Fig. 9d–f, assuming that the axons on the intervals roughly had a rectilinear shape. The values denoted by an asterisk are the widths measured using the CLM images of Fig. 5, which correspond to the lengths when θ = 0°. The result implies that the axons grew up to over 200 μm of the lengths, and connected respective cells on neighboring two deposited areas.

**Figure 10:**
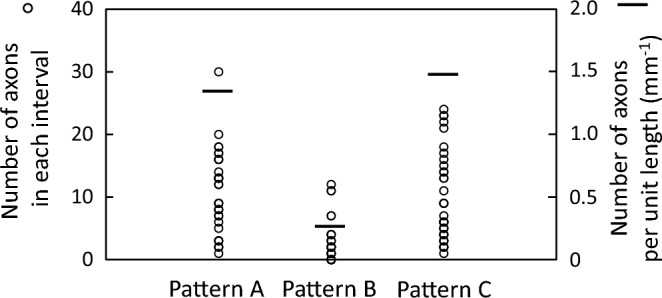
The numbers of axons on the intervals counted using the images over the whole surface of each substrate as for Pattern A, B and C. The left-hand and right-hand vertical axis represent the number of axons in each interval (○) and the number of axons per unit length of the interval (—), respectively.

The numbers of axons connecting on the intervals were investigated using the images photographed over the whole surface of each substrate as for Pattern A, B and C, and shown in Fig. 10. The left-hand vertical axis represents the number of axons in each interval between neighboring DLC deposited areas (see Fig. S4), and one open circle corresponds to the number of axons counted at one interval. More, because the lengths of the DLC deposited areas in the longer direction were different by the patterns, values obtained by dividing total numbers of axons by the sum of lengths of all intervals are shown as a horizontal line with the right-hand vertical axis. It is highly probable that the difference in the lengths on the deposited areas arose from the presence or absence of stretching PDMS substrates and difference in its direction. It is noted that obvious difference in the number appeared by the patterns, and the number of Pattern A and C were considerably larger than that of Pattern B. Pattern B allowed the axons to stretch with higher orientation on the deposited areas as described, and it is implied that the axons were less subject to move outside the deposited areas than the others, resulting in fewer connections.

From the results in Figs. 5, 6, 7, 8, 9, 10, the feature of each pattern can be summarized as stated below. Pattern B was characterized by the structure that more axons were unidirectionally arranged on the DLC deposited rectangular areas, and the aggregations of aligned axons were separately placed at intervals and connected by many axons each other. In Pattern C, the aggregations of axons connected in mainly parallel and perpendicular directions on the rectangular areas were positioned in regular intervals, and much more axons connected the aggregations to each other. More, Pattern A shared features of both Pattern B and C, exhibiting that the aggregations formed with axons arraying relatively unidirectionally were connected by the axons of amount comparable to Pattern C. The connecting axons in any pattern were two-dimensionally formed on PDMS surface and individually taut in a straight line at about 100 to over 200 μm in length.

## Conclusions

In this study, the DLC thin films were deposited on PDMS substrates using the metal mask by the plasma CVD method, and on the fabricated DLC/PDMS substrates human neuroblastoma cells, SH-SY5Y, were cultured to evaluate the cellular behaviors. The major results are summarized as follows. Three kinds of distinctive interconnection structures of axons were created on plane PDMS substrates partially DLC-deposited in a rectangle shape. These substrates had the design patterns that 50 rectangular areas with respective dramatically different wrinkle structures were adjacently arrayed in regular intervals. In any case, the structures possessed a number of individual stretching axons, which were two-dimensionally formed on PDMS surface and taut in a straight line one by one at about 100 to over 200 μm in length. This demonstrates that plenty of independent taut axons on the fabricated substrates can be applied to various evaluations relating to axon behaviors in vitro, and the functions are available without setting up guiding grooves generally used in microfluidic devices. Therefore, the methods proposed in the study can be performed without depending on conventional soft lithographic technologies requiring multiple stages and their treating times. In addition, absence of a top cover essential to driving of microfluidic devices creates greater flexibility for the observation and various evaluations of neuronal cells cultured.

## Supplementary Information

Supplementary Figures.

## Data Availability

The datasets generated during and/or analyzed during the current study are available from the corresponding author on reasonable request.
