# Supplementary material for: Fabrication of plane-type axon guidance substrates by applying diamond-like carbon thin film deposition
Source: Sci Rep. 2023 May 25;13:8489. doi: 10.1038/s41598-023-35528-3 (PMC10213005; doi:10.1038/s41598-023-35528-3)
Supplement: Supplementary file 1 — Supplementary Figures. [file 41598_2023_35528_MOESM1_ESM.pdf]

Supplementary Information

Fabrication of Plane-type Axon Guidance Substrates by Applying Diamond-like Carbon Thin Film Deposition

Authors: Masahito Ban, Jing Chen

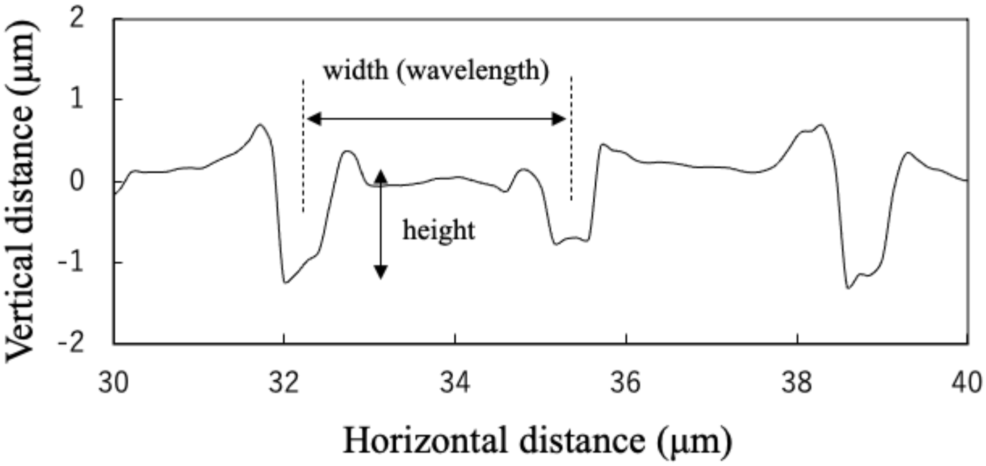

Fig. S1 Typical cross-sectional profile of wrinkles measured by CLM as for the sample at the strain of 0.2 and the deposition time of 2 min in Test A. Using the data of the horizontal distance of 60 μm, the average width (wavelength) and height of the wrinkles were obtained as about 3.0 and 1.2 μm, respectively.

The wave-like pattern can be explained by a model that as for a thin, stiff coating film on a thick, soft substrate, the compressive stress arisen from the mismatch of both elastic moduli causes buckling of the film [23-25]. Using the equations (1) and (2) according to the model, the width and height of wrinkles were estimated to be 1.8 - 2.3 and 0.80 - 1.0 μm, respectively. Where λ and *h* are the width and height, and *E* and *e* are Young's modulus and the applied external strain, respectively. *t* is the DLC thin film thickness. Here the subscripts *f* and *s* refer to the DLC thin film and PDMS substrate, respectively. Typical values of Young's modulus (DLC thin film, E_f = 50 - 100 GPa and PDMS, E_s = 2 MPa) and the DLC thin film thickness of 14 nm were used for the calculation.

$$\lambda \approx 4.4t\left(\frac{E_f}{E_s}\right)^{\frac{1}{3}} \quad (1)$$

$$h \approx \lambda e^{\frac{1}{2}} \quad (2)$$

The compressive stress σ_1 (Pa) by thermal expansion is given by equation (3) at temperature T below the deposition temperature T_D:

$$\sigma_1 = \frac{E_f(\alpha_s - \alpha_f)(T_D - T)}{(1 - \upsilon_f)} \qquad (3)$$

Here, ν is the Poisson's ratio, and α (°C^{-1}) is the coefficient of thermal expansion. Using the parameters for DLC and PDMS: $\nu_f = 0.22$, $\alpha_f = 5 \times 10^{-6}$ °C^{-1}, and $\alpha_s = 3 \times 10^{-4}$ °C^{-1}, given that the temperature increase of the substrate was up to about 100 °C during deposition, σ_1 is calculated to be 1.4 - 2.8 GPa.

In the case of stretched PDMS, the compressive stress σ (Pa) is induced by not only the thermal expansion and shrinkage during the deposition process but also the stretching in advance and removal. The compressive stress σ_2 (Pa) by PDMS stretching is estimated by equation (4):

$$\sigma_2 = \frac{E_f(\varepsilon_0 - \varepsilon_r)}{(1 - \upsilon_f)} \qquad (4)$$

Here, ε_0 is the strain firstly applied and ε_r is the remaining strain after unloading. Using $\varepsilon_0 = 0.2$ and $\varepsilon_r = 0$ (in the case of completely elastic deformation), σ_2 is calculated to be 13 - 26 GPa. The stretched PDMS substrate was not actually restored to the former state by the unloading, and the calculated σ_2 indicates the maximum values. When σ is simply the sum of σ_1 and σ_2, it becomes about 14 - 29 GPa.

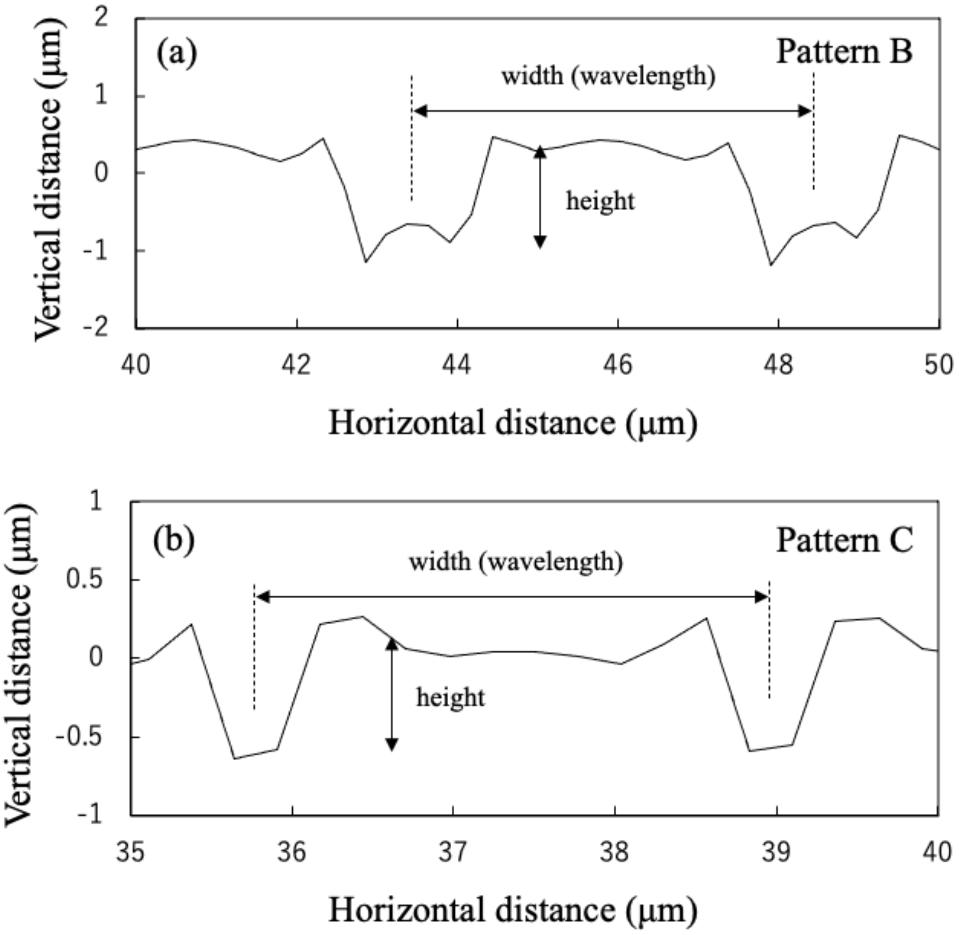

Fig. S2 Typical cross-sectional profile of wrinkles measured by CLM as for (a) Pattern B and (b) C in Test B. Using the data of the horizontal distance of 80 - 100 μm, the average widths / heights of the wrinkles of Pattern B and C were obtained as about 5.1 / 1.3 and 3.2 / 0.7 μm, respectively.

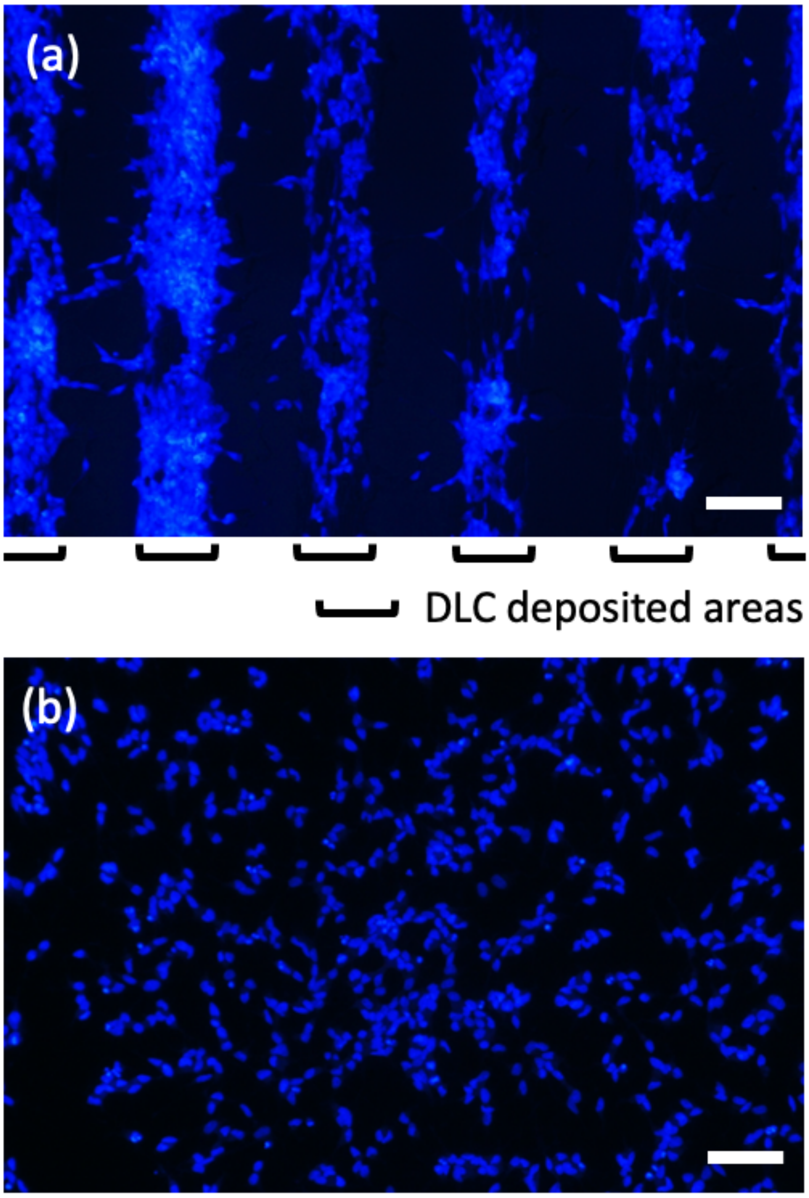

Fig. S3 The typical images obtained from fluorescent microscope observations: (a) Pattern A and (b) no strain of Test A (without the metal mask). Cell nuclei stained in blue with DAPI. Scale bar corresponds to 100 μm. Seeing (a), the nuclei are found to gather in a portion of the surface where the holes of mask existed, meaning that the cells attached selectively on the DLC deposited wrinkle areas. It may be suggested from our former study [27] and recent experiments that the phenomenon occurred due to higher protein adsorption property of a DLC thin film compared to PDMS used as the substrate. As the reference, as can be seen in (b), the nuclei stained in blue were scattered all over the substrate surface.

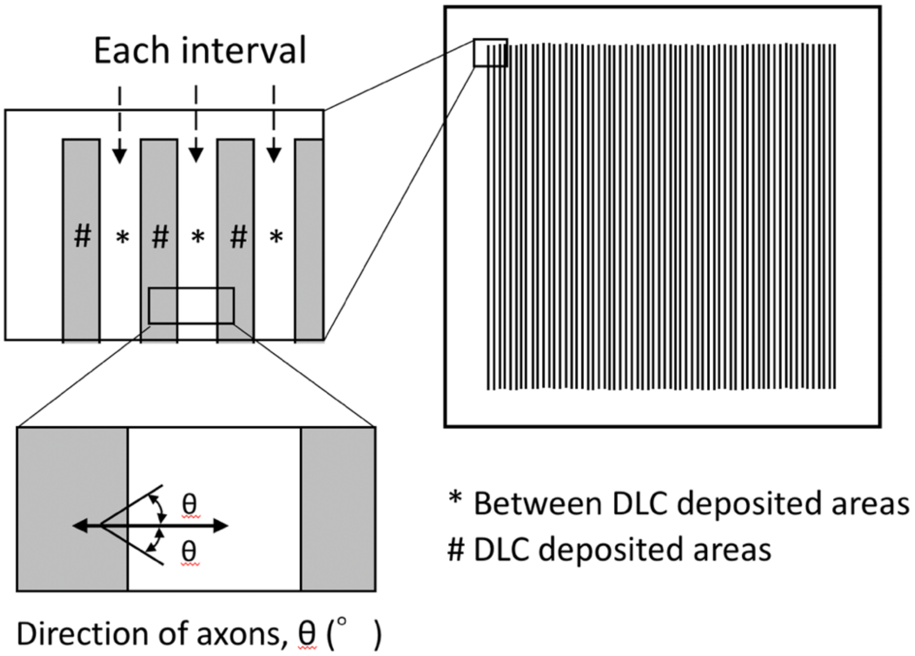

Fig. S4 The schematic diagram of the DLC deposited PDMS substrate. The direction of axonal elongation on a substrate is defined in the figure. That is, θ denotes the angles from the direction (0 °), which is perpendicular to the longer direction of the DLC deposited rectangular areas. Concerning the cells on both the DLC deposited areas (# in the figure) and the intervals between the deposited areas (* in the figure), the stretching directions of axons were evaluated in Fig. 9.

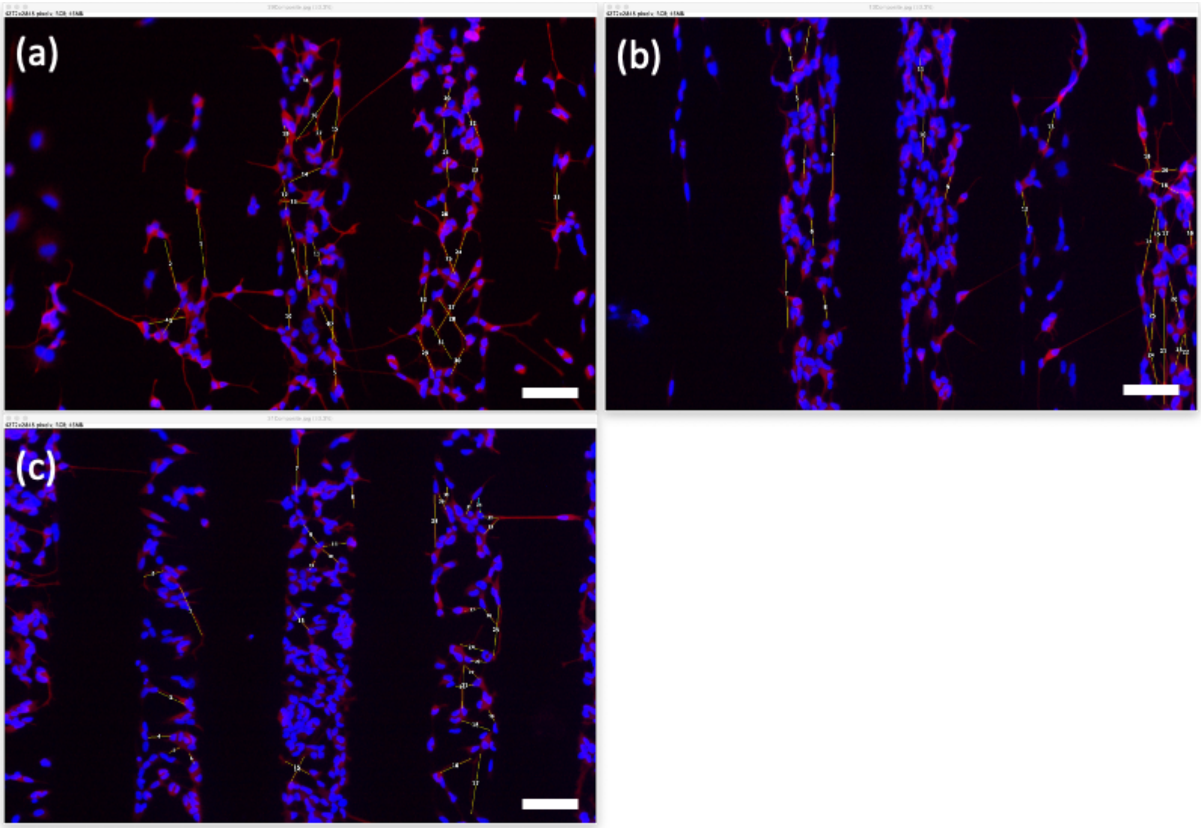

Fig. S5 Typical images of the result obtained when the directions of axons were measured using ImageJ software, as for cells on the DLC deposited areas ((a) Pattern A, (b) B and (c) C)**.** Using the merged images of Tuj1 (red) and DAPI (blue), cells having clearer outgrowths were extracted, and each direction of axons extended from cell body was measured. Scale bar: 100 μm.

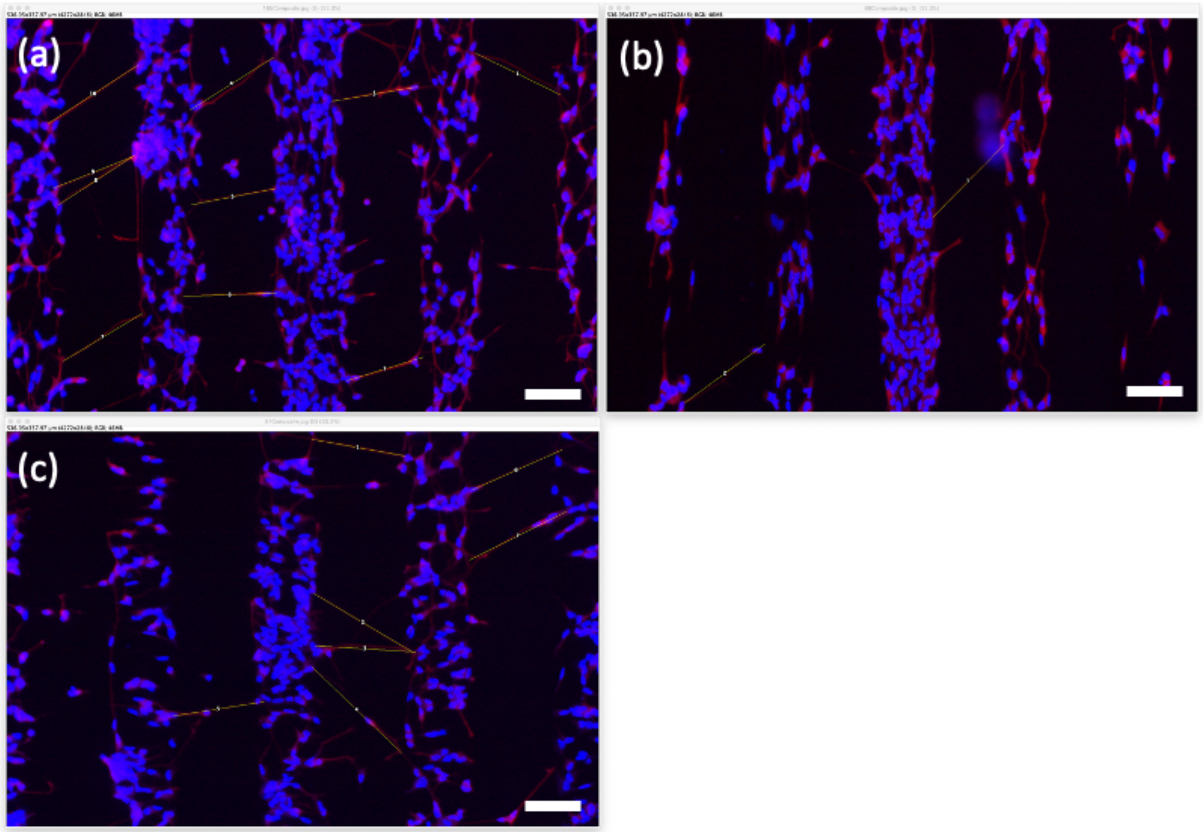

Fig. S6 Typical images of the result obtained when the directions of axons were measured using ImageJ software, as for intervals between the DLC deposited areas ((a) Pattern A, (b) B and (c) C). Using the merged images of Tuj1 (red) and DAPI (blue), axons connecting neighboring DLC deposited areas were extracted, and each direction of axons was measured. Scale bar: 100 μm.
